# Rethinking retention: Mapping interactions between multiple factors that influence long-term engagement in HIV care

**DOI:** 10.1371/journal.pone.0193641

**Published:** 2018-03-14

**Authors:** Stephanie M. Topp, Chanda Mwamba, Anjali Sharma, Njekwa Mukamba, Laura K. Beres, Elvin Geng, Charles B. Holmes, Izukanji Sikazwe

**Affiliations:** 1 Centre for Infectious Disease Research in Zambia, Lusaka, Zambia; 2 College of Public Health Medical and Veterinary Sciences, James Cook University, Townsville, Australia; 3 Bloomberg School of Public Health, Johns Hopkins University, Baltimore, Maryland, United States of America; 4 School of Medicine, University of San Francisco, San Francisco, California, United States of America; Médecins Sans Frontières (MSF), SOUTH AFRICA

## Abstract

**Background:**

Failure to keep people living with HIV engaged in life-long care and treatment has serious implications for individual and population-level health. Nested within a four-province study of HIV care and treatment outcomes, we explored the dynamic role of social and service-related factors influencing retention in HIV care in Zambia.

**Methods:**

From a stratified random sample of 31 facilities, eight clinics were selected, one urban and one rural from each province. Across these sites we conducted a total of 69 in-depth interviews, including with patients (including pregnant women) engaged in-care (n = 28), disengaged from care (n = 15), engaged facility transferee (n = 12), and friends/family of deceased patients (n = 14). At the same sites we conducted 24 focus group discussions with a total of 192 lay and professional healthcare workers (HCWs). Two-day observations in each of the eight facilities helped triangulate data on operational context, provider relations and patient-provider interactions. We ordered and analysed data using an adapted version of Ewart’s Social Action Theory.

**Results:**

Three overarching findings emerged. First, the experience of living with HIV and engaging in HIV care in Zambia is a social, not individual experience, influenced by social and gendered norms and life goals including financial stability, raising family and living stigma-free. Second, patients and their networks act collectively to negotiate and navigate HIV care. Anticipated responses from social network influenced patients’ willingness to engage in care, while emotional and material support from those networks influenced individuals’ capacity to remain in HIV care. Lastly, health system factors were most influential where they facilitated or undermined peoples’ collective approach to health service use. Participants living with HIV reported facilitation of both their initial and continued engagement in care where services involved social networks, such as during couples testing and community outreach. Conversely, service features that were poorly aligned with respondents’ social reality (e.g. workplace obligations) hindered long-term engagement.

**Conclusions:**

This study moves beyond listing barriers or socio-ecological groupings, to explain how social and health systems interact to produce HIV care outcomes. Our findings challenge the implicit assumption of individual agency underpinning many retention studies to highlight the social nature of illness and healthcare utilization for HIV in Zambia. This understanding of collective action for accessing and remaining in HIV care should underpin future efforts to revise and reform HIV and potentially other chronic service models and systems.

## Introduction

Promoting long-term engagement in chronic care in Low and Middle Income Countries (LMIC) is a growing priority for national and global health programmers [1, 2]. As populations age, more people in LMIC than in rich countries face disability, morbidity and mortality due to chronic diseases [3, 4]. These undesirable outcomes occur because few people in LMIC are screened for chronic conditions and remain engaged in life-long treatment [3, 5]. In sub-Saharan Africa, health systems are further challenged to diagnose and retain a growing number of patients on anti-retroviral therapy (ART) [6, 7]. Failure to keep people living with HIV engaged in life-long HIV care and treatment has serious implications for national health and productivity [5, 8–11].

Zambia has approximately 1.2 million people living with HIV, two-thirds of whom have started ART. In 31 healthcare facilities, intensive tracing of a stratified random sample of patients who were at one time engaged in care found that 86% of persons living with HIV retained in care after 12 months and 78% after 24 months of ART initiation [12]. Alarmingly, 22% had died within two years of ART initiation [12]. The 3,257 sampled patients confirmed as disengaged from HIV care reported structural, psychosocial and clinic level barriers that greatly varied by clinic [12]. Zambia must address these specific structural, psychosocial and clinic level barriers in order to achieve the ambitious 90-90-90 goals [6] which can help to prevent premature death and achieve national socio-economic and development goals [13].

We conducted an explanatory qualitative study in order to better understand the interactions between these factors driving engagement or disengagement from care. For the purposes of this study, we define ‘engagement in care’ broadly, as the complete set of decisions and actions necessary to ensure an individual has access to, and is both capable of and willing to act on medical advice, including taking their medications. Although we view ‘adherence’ and ‘health seeking’ as more narrow subsets of ‘engagement in care,’ we acknowledge that others [14–16] have chosen to define these concepts differently. Similarly, various theories and models exist to help understand the complex and interacting factors that influence engagement in health care [17, 18], albeit with substantial conceptual overlap. Nonetheless, much of the HIV adherence and engagement-in-care literature to date rest on opaque assumptions regarding the influence of social or organizational context on individual motivations (a mediator) and actions (such as engagement in care). In particular, detailed explanatory accounts on the influence of social and organizational context on individual behaviours in LMIC settings are limited, albeit with some notable exceptions [19, 20].

**Fig 1:**
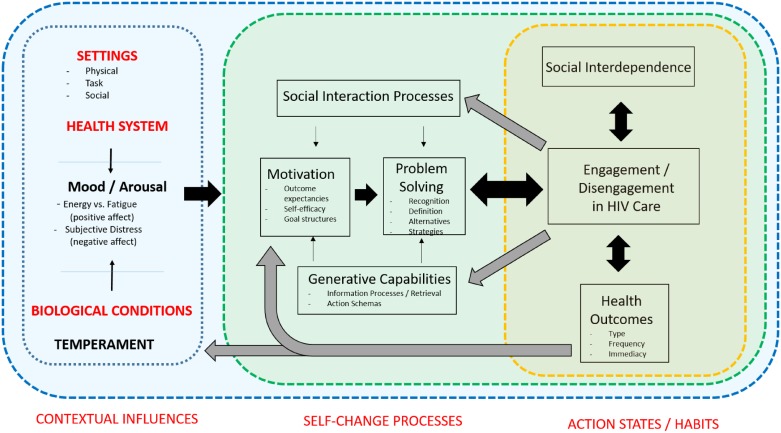
Adapted Social Action Theory framework.

In this paper, we use an adapted version of Ewart’s Social Action Theory (SAT) [21] to explore the relationship between broader social and organizational influences and individual motivations and circumstances driving engagement or disengagement in HIV care (Fig 1). Ewart [21] suggests that health behaviours (such as engagement in care) are the result of an interplay between three domains. The first domain, ‘action contexts’ includes the broader socio-cultural and socio-economic setting, organizational settings (such as health services), as well as contexts specific to the individual (such their temperament and physiological state). The second domain ‘processes of self-change’ include an individual’s generative capabilities, such as how they process and make sense of information; an individual’s social interactions with family and networks; mechanisms of motivation such as self-efficacy, expectations of certain outcomes, and life goals; and finally problem solving capabilities. Together action contexts and processes of self-change influence the third domain, or ‘action state’ in which the behaviour of interest (in this case, engagement or disengagement from HIV care) takes place, with feedback loops generated by the outcomes of the action.

A strength of SAT vis-à-vis complex behaviours such as engagement in HIV care, is that it seeks to make explicit the relationship between the broader economic, socio-cultural and organizational contexts in which actors live and work, and the more proximate individual level mechanisms (e.g. motivation or problem solving) that catalyse care-engagement decisions. By empirically studying the relationships outlined in Fig 1, we therefore seek not only to improve the evidence base but also to develop the field, demonstrating how a more rigorous, theoretically grounded explanatory account can inform robust policy and program reform to improve long-term engagement in chronic care in low-income settings.

## Methods

This explanatory qualitative study drew on the larger study conducted among a stratified, random sample of 4,362 patients identified as lost to follow-up (LTFU) in 31 health care facilities using electronic medical records (EMR). LTFU was defined as having had a recorded HIV-related visit at the facility in the past two years followed by a gap in EMR-recorded visits of ≥90 days since the last scheduled appointment. The goal of the larger study was to ascertain HIV care and treatment outcomes categorized among these ‘lost’ patients as i) currently in adult HIV care at their original clinic; ii) disengaged from care, iv) in HIV care after transfer to a different clinic and, v) deceased. Nested within that, we conducted this qualitative enquiry to answer questions about how and why PLHIV are engaging or disengaging in care. Operationally, the category of ‘engaged in-care’ patients in this study included those who reported having visited a health facility for HIV care in the 3 months prior to being asked.

We conducted a total of 69 in-depth interviews (IDIs). These included 28 with people living with HIV and currently receiving care and treatment at their original clinic, 15 disengaged, and 12 transferred to understand barriers and facilitators of engagement; and, 14 IDIs with family members or close friends of patients who were confirmed deceased in order to gather their perceptions of events leading to death. Twenty-four (24) focus group discussions (FGDs) with professional HCWs and lay staff sought insights into their perceived role in patient engagement and ability to fulfill that role. Lastly, two-days of non-participant observation were conducted by pairs of trained research assistants, in eight health care facilities, in public (waiting area) and private (consultation rooms) areas; this data enabled triangulation of information on operational context, provider relations and patient-provider interactions. The total data set comprised transcripts from 69 IDIs and 24 FGDs and memos generated from observations in eight health care facilities.

### Site selection

Eight health care facilities were purposively selected from the 31 sampled facilities in the larger study, to include one urban health centre or district hospital, and one rural health centre from each of the four provinces. After completing the first round of data collection at these eight facilities, an additional four facilities were purposively selected by the first author in consultation with other investigators to conduct FGDs that tested initial findings and probed emerging and unclear issues; these facilities included two urban and two rural sites, one from each province.

### IDIs’ sampling and data collection

In each of the original eight health care facilities, we interviewed four to five ‘in-care’ patients, including two pregnant women, using simple-random sampling of files of HIV patients present that day. If a sampled patient declined an interview, the team selected the next file. We also conducted two IDIs per clinic in each of three categories of ‘lost’ patients, i.e., disengaged, transferred or dead. Disengaged and transferred patients successfully found by a peer educator by phone or in person by the larger study, were immediately contacted for a follow-up interview. For those confirmed dead, a family member or close friend was invited to an IDI. Although we made an effort to interview a man and a woman per ‘lost’ category, this was not always pragmatic or possible. No patients from those sampled in the clinic (e.g. pregnant and in care) refused to participate. Of the ‘lost’ patients sampled through tracing, no patients refused to participate in the interviews at the final stage of recruitment when full information and consenting was conducted. At the earlier stage of recruitment, four (out of a total of 44) traced respondents declined to provide their details to receive a follow-up phone call or visit to discuss the possibility of an interview; the remaining 41 provided details and ultimately consented to participate.

Interviews were conducted by experienced research assistants (RA) recruited for their ability to conduct interviews in multiple languages. RAs received a one week of training including refresher interview training, specific training on the study intent and interview guides, and in human subject protection. IDIs lasted between 40–120 minutes and were conducted in the participant’s choice of English or local languages Nyanja, Tonga, Bemba, or Lozi. Open-ended interview questions enabled in-depth discussions to probe for emerging themes, new experiences, and potential causal mechanisms influencing patients’ engagement in care. Questions guided participants to reflect on their (or their family members’) perceptions, choices and behaviour in relation to HIV care-seeking history; and their experiences negotiating the current health system, including the way information availability, socio-cultural norms, and HCW attitudes, have affected past and current health seeking behaviour.

### FGDs’ sampling and data collection

In each of the 12 facilities, we conducted one FGD with professional HCW and a separate FGD with lay staff to facilitate free discussion. Recruitment was through open invitation on a ‘first come, first served’ basis, with a minimum of four participants in each group. FGDs ranged from 1 to 3.5 hours. Though dependent on participant preferences, FGDs were usually conducted in English with professional health staff and in local languages with lay staff. FGDs were facilitated by a trained facilitator using an open-ended FGD discussion guide on barriers and facilitators to patient engagement in care including HCWs’ perception of their role in this. We further probed discrepancies between HCWs’ perceptions and patients’ described experiences within health facilities.

### Direct observation procedures: Non-participant observations

Data were collected over two days, by the same research assistants who conducted IDIs, during multiple two-hour non-participant observation sessions in (public) clinic waiting areas and (private) clinical consultations with both patient and provider consent. Hand written notes were taken on semi-structured observation templates, and subsequently transcribed into electronic memos. Data collected during observations contributed to building a picture of typical workflows and human interactions that drive health centre operations and that influence patients’ experience and decisions related to care-seeking [22]. These data also helped supplement structured health centre audits through various informal conversations and fact checking opportunities.

### Data management and analysis

All procedures were subject to written informed consent including to audio-record IDIs and FGDs. Participants did not use their own name during FGDs and were issued with a participant number or used a pseudonym. All audio-recordings were translated to English (where necessary) and transcribed verbatim. Transcripts, observation memos and notes were imported into NVivo QSR^™^ and subjected to an iterative process of coding. Analytical categories of related codes and sub codes were stratified by study sites and participant profile to be exported as tables in word processing software for synthesis and analysis on health systems factors influencing patients’ decision to remain engaged, disengaged or transfer out of clinic for HIV care. A draft code book developed with reference to SAT and refined following a first reading of the transcripts using deductive reasoning, was continually updated during the iterative coding processes using inductive reasoning to ensure the inclusion of emergent themes.

### Ethics

The University of Zambia Biomedical Research Ethics Committee (UNZABREC) and University of California, San Francisco (UCSF) Institutional Review Board (IRB) approved the study.

Limitations: As a qualitative study, our findings are neither quantifiable nor directly generalizable. By its very nature, our enquiry considers both rare and more frequent phenomena rather than look for association, causation or statistical significance. However, we collected data from a large number of patient and health workers from diverse settings—urban and rural sites in four provinces in Zambia to capture the range of experiences influencing care seeking by people living with HIV. Deep and authentic engagement with participants could result in their over- or under- stating the circumstances leading to their action states. Audio-recording for tone of voice and field notes for non-verbal communication attempt to guard against these pitfalls. Further, we triangulated data against those generated from direct (non-participant) observations. Thus, while specific findings cannot be generalized beyond the four Zambian provinces in which the study occurred our analysis plays to the strength of qualitative research to learn from participants how they experience and make sense of events within a particular setting and processes. Our openness to serendipity allowed us to fit theory based on the emerging data. This grounded approach led to the choice of SAT to capture the complex web of factors within which care seeking and engagement in care occurs. In applying SAT we hope to extend the analytical generalizability of this work, but recognize this paper as a first step on which future work can build to strengthen the application of such theories relevant to the domain of retention research in LMIC contexts, the methods used and the domains can be transferred to the country and other settings.

## Findings

We present findings according to the three major domains from the Social Action Theory framework (Fig 1); namely i) action settings ii) self-change processes and iii) action states. Findings from both patient and HCW data are supported by illustrative quotes from patients themselves, where available. Quotes from HCW reflect findings that did not align with and reinforce patient views, or where HCWs provided extra emphasis or additional information. Interpretation and consideration of the theoretical implications and fit of the data, as a whole, was a continual process of coding, comparison and triangulation from all sources of data.

### Action settings

#### Physiological state

An important action setting for many PLHIV was their own state of physical health. Prior experience of physical decline or recurrent illness was an important backdrop to the decisions of many (n = 51) to seek or stay in care:

I used to get sick in the past but I didn’t believe that I was HIV positive. But then it’s because that I used to get sick all the time, that is why I realized that it’s better I go and get tested.

[Rural Male, Eastern, In Care]

OK, the main reason I started [ART] is that I got very sick. Because I couldn’t even walk a short distance from here to there [pointing a few metres away]. I was even failing to lift a bucket of water.

[Rural Female, Southern, In Care]

It made it easy because I was sick at the time. How can I explain it? It made it easy because I was sick at the time, so it made me start ART. The way I was is what made me to start ART.

[Rural Female, Southern, Transfer]

#### Social setting

Findings pointed strongly to the critical role of social setting of persons living with HIV as an action setting influencing decisions regarding engagement in care. Here we define social setting in a broad sense, encompassing social and familial networks, livelihoods and cultural practices.

More than half the respondents reported that they felt their communities ‘accepted’ people living with HIV:

They do accept because in the community, [I] am not the only one, nor is it the first time this is happening.

[Urban Female, In-Care, Eastern Province]

Despite this, a recurring theme in respondents’ accounts was the experience of felt or anticipated stigma. Some described instances of overt stigmatization including branding of HIV-infected individuals as ‘promiscuous’.

When you tell someone that you have a HIV virus they would start thinking that you have been promiscuous. And then they would start telling others that she was a prostitute and she picked HIV from that. That is the reason why I haven’t told anyone except that neighbour.

[Urban Female, Pregnant In-Care, Eastern Province]

Many more described fearing being gossiped or talked about behind their back and the influence that this had on their decisions to disclose:

Yeah, because you see in our communities, if somebody knows that you are HIV positive, there are so many, I mean, rumours which goes around! So it’s better you keep it, as you have closed the door, we keep it confidential. So, it’s confidential between myself, and my cousin and my wife.

[Urban Male, In-Care, Lusaka Province]

People living with HIV commented that in order to improve community acceptance, greater effort had to be made in helping to educate HIV-negative members of the community and address normative responses to HIV.

The things that can make [the community] more accepting is if people from the community could be helped to understand.

[Rural Female, Disengaged, Lusaka Province]

Let me just say that a lot of information is still not there, and what is needed is for you to encourage people with information. Understanding this thing [HIV], it helps.

[Rural Female, Western, In Care]

#### Health systems and service organization

Issues of health service access, cost, efficiency and quality of experience all emerged as contextual influences (positive and negative) on individual decisions regarding HIV care. Some factors were related to the health system generally and others specifically to HIV service delivery.

#### Health system setting

Patients frequently noted that free provision of HIV care and treatment was an important structural facilitator of their access to and engagement in care.

It was easy [to be in treatment] because firstly that medicine is not bought, but [is] for free. Enrolling is free […] They just encourage you, come for free.

[Rural Female, In Care, Southern Province]

However, cramped infrastructure leading to overcrowding and lack of privacy for basic consultations was reported as a concern among some patients, and confirmed by many health workers. Lack of space and privacy leading to involuntary disclosure and community gossip emerged as an important influence on patients’ assessment of the service context as described below:

When an individual goes to the clinic, there are people […] They will come to see what goes on there at the ART clinic. So they can come and tell people what is happening. And that is what makes me feels lazy.

[Rural Female, Disengaged, Eastern Province]

Distance to the clinic, and associated time and cost were mentioned by many participants—particularly rural respondents. Representing a critical interaction between social and health system action settings, distance was also a challenge for patients who chose to enroll in health centres outside their own residential area in order to preserve confidentiality; or for those who had to move or travel for family or work reasons but without means to pay for regular return transport.

I just didn’t stop for the sake of stopping but I had encountered a problem. I never had money to travel back and where I was working they never paid me on time. So I failed to travel back.

[Rural Female, Disengaged, Western]

I transferred from [my first clinic] because I divorced my husband who lived there. So it became far for me, to come from here, going to back there. That is what made me to transfer from there to this mission here.

[Rural Female, Transfer, Southern]

A large number of respondents (n = 49) described health workers as the most likely source of trusted information about HIV and HIV care and treatment. Respondents’ accounts—even some disengaged patients—emphasized that health workers often improved their understanding and ability to cope with their diagnosis and treatment by providing ‘education’ and helping ‘teach’ patients about their disease.

The benefit is there because I have learnt a lot from [the staff]. The first thing they taught us was how to live, how to eat and how to keep ourselves.

[Urban Female, Transfer, Lusaka Province]

A number of patients described HCWs playing an important role in encouraging them to think positively about their diagnosis and providing them with counselling and emotional support.

I think I can only give my gratitude because when I got there and explained to them, they never took me in any other way but they advised me and encouraged me a lot to say: “Don’t go backwards.”

*[*Urban male, Disengaged, Western Province]

Notwithstanding these points, the chronic lack of HCWs, and associated queues and waiting times, were frustrating aspects of the health service context frequently reported by participants.

Interviews also revealed that recurring perceptions of HCWs as unprofessional—in the sense that they were often not timely, lacked attention to detail, failed to meet standards of confidentiality, displayed disrespect and were abusive—contributed to often negative perceptions of the health service context.

Ah! they care rudely! They don’t care as you are taking care of me right now, if at all. I wish it was you who was there! They care rudely!

[Urban Male, In Care, Lusaka]

When you go [to the clinic] and ask, they would shout at you. But they are not supposed to shout at [us]. Instead they are supposed to encourage that person […] Just to say the truth, one of the reasons why I stopped care is because, there, they shout at us very much, they are rude.

[Urban Female, Disengaged, Eastern Province]

#### HIV treatment service setting

Positive aspects of HIV-specific services noted by persons living with HIV included the expansion of some rural services—via mobile services, integrated services, or increased number of ‘ART-days’–improved their ability to access and remain in care. A range of respondents—men and women—commented on the positive experience they had had with couples testing, adherence counselling (particularly for side effects), general education talks, and (less commonly) follow-up services, which buoyed clients’ mood.

It was easy [to enroll]. I had come with my husband to test, that is how they found us both positive and I started treatment.

[Rural Female, Pregnant In Care, Lusaka]

Despite the many concerns relating to stigma described above, some respondents described the clinic as a setting in which they accessed general emotional or psychosocial support from other HIV-positive individuals. Patients also described the rigour of HIV-care processes, including having weight and temperature measured and blood tests, as an important factor in their long-term engagement in care (Box 1) via patients’ sense of trust in health worker and the health system competency.

**Box 1: Alternative treatments**

Eight individuals (12%) agreed they (or their relative) had sought help from an alternative provider at some stage, however a large majority (88%) of patient-respondents denied having sought or used non-Western medicines. In explaining why, several themes emerged.

**Clinical Efficacy**: patients described health centre processes as more rigorous than those used by traditional healers. Comparisons focussed on the health centres’ use of equipment (blood pressure cuffs, thermometers) and blood tests to establish baseline and ongoing health status as well as the provision of education and information by health care staff.

It is different [with a traditional health] because that one won’t explain to you what is paining you, or how it will work with the medicine he will give you. [Rural Female, Transfer, Southern Province]

**Efficacy of ARVs**: participants routinely compared ARVs to ‘weak’ traditional medicines. Although some suggested that traditional medicines ‘might have worked in the past’ or may still work for other conditions, many noted that they were not ‘strong enough’ for HIV.

These [ARVs] are working. I have not gone to another place for a traditional healer because that medication doesn’t do anything. As for the traditional medication it doesn’t work nowadays, [although] those old days the traditional medication use to be very effective. [Urban Female, In Care, Eastern Province].

**Cost**: many commented that treatment at the health centre was free (and effective) while traditional healers just ‘took your money’.

With the traditional healer there are times when you spent money which you can’t even see. [Urban Male, In Care, Eastern Province].

Mmmmmm the traditional healer can only finish your money. But at the clinic there’s nothing paid, it’s free of charge. [Urban Male, Disengaged, Western Province]

While alternative or concurrent use of traditional medicines was described, therefore, the overall picture presented by respondents in this study suggests that the provision of antiretroviral drugs via free public health centres is increasingly considered the more trustworthy and effective option by a most Zambians.

However, a recurring theme in respondents’ descriptions of barriers to care was the inflexibility of HIV visit schedules and associated medication refills. Nearly half the patients mentioned these schedules, with experiences ranging from inconvenience to fundamental clashes with family or work commitments.

They give three months [of drugs] but you have been taking the medicine for three years. So you find that you are knowledgeable and are in line with the medicine without any reactions. So at least if they would put/give for six months […] so that we can stay for a long period. It would help.

[Urban Male, In Care, Southern Province]

Like I have explained, I am a bricklayer. Once or even three times I told them: “I am going to work and I don’t know when I can come back. The one month [of drugs] you are giving me, its better you give me for 3 months.” But they refused, saying: “It’s our program, so it’s better we give you something for 1 month.” So I tend to think that these people have not thought about me.

[Rural Male, Transfer, Eastern Province]

In more than one instance, participants described being forced to choose between fulfilling family care duties or obligations and accessing care, due largely to scheduling constraints within the health system:

My husband was sick, so I was the one used to come and get the drugs for him and for myself. So our dates started differing mine and his. […] things were too much for me that is how I stopped.

[Urban Female, Disengaged, Lusaka Province]

Compounding these frustrations was the guideline-mandated response to any recorded gap in treatment; a period of ‘intensive adherence counselling’ requiring at least three adherence sessions that could be done over several weeks to collect medications and receive counselling. Health providers in all four provinces stressed that this measure was about impressing on patients the need to take their drugs on time. Despite this, various patients noted that intensive adherence counselling merely exacerbated the tensions between their treatment and work or family arrangements, sometimes forcing a tradeoff.

I stopped going there [the clinic] because they gave me a lot of [scheduled visit] days … so I thought that let me just stop!

[Urban Female, Disengaged, Western Province]

### Self-change processes

#### Generative capabilities

Patients’ knowledge and understanding of HIV and ART emerged as an important factor influencing decision processes relating to engagement in care. In fact, persons living with HIV and healthcare workers in all four provinces emphasized the need for better coverage of health education and counselling to improve clients’ and communities’ understanding of HIV treatment.

Key concerns included the role that lack of knowledge played in fueling fear and misconceptions related to HIV treatment:

Before I started ART, I was in the dark. I didn’t know what it was […] I used to get upset when someone comes to me and asked about me going to the clinic.

[Urban Male, Western Province, In Care]

I was scared because people say that when [you] start taking ARVs, you will have dizziness and there are people who get confused when they take them. As a result of that, I was a bit scared.

[Rural Female, Eastern Province, Transfer]

Lack of knowledge was also linked to decisions to disengage from care due to the belief symptom alleviation was the same as cure:

It was the issue of ignorance. After taking [ARVs] I got better and I didn’t know that I had not yet been cured

[Rural Male, Eastern Province, Disengaged]

Further, participants’ association with their HIV status influenced their motivation to remain in care. The process of accepting one’s diagnosis as accurate was rarely straightforward as described in the quote below:

I was not feeling well last year. The whole of last year I was not feeling well […] so I thought: “let me get tested.” I went to the hospital and there they found me positive. But I did not get treatment. I denied it. So I came to Lusaka. When I came to two places to get tested, they found me positive, but again I refused. I went to one clinic, then another to visit my sister [a nurse], she tested me again and I was positive. So that was the third time. So I just agreed finally they can’t all lie. So finally I went to the clinic.

[Rural Female, In Care, Lusaka Province].

Further influences on participants’ acceptance included the role of alternative treatments and religious beliefs. A summary of findings relating to each of these topics are outlined in Boxes 1 and 2 respectively.

**Box 2: Religious influences**

A majority of respondents (74%) in this study reported being current church goers while almost all reported having attended church at some point in their lives. Consistent with our finding that it was unusual for patients to disclose their HIV status outside the family, most respondents reported they had not told members of their church. While only a very few participants described treatment decisions being actively influenced by their Church, however, a number did note that the influence of Pentecostal churches (versus mainstream Christian denominations such as the Catholic Church) in encouraging patients to stop treatment and ‘trust in prayer’ or other faith cures.

The church where I worship is the Catholic [church]. We don’t have any problem. The people who might have a problem are those from the Pentecostals. Because there when you give the testimony that you are HIV positive they would want to pray for you so that you can stop taking the drugs […] they confuse the patients [Urban Male, In-Care, Eastern Province]

Importantly, however, accounts of faith-driven actions were linked to patients’ decision to both engage in, and disengage from care.

Zambia being a Christian nation […] we have seen a lot of people going for prayers. Even now if someone announces prayers are taking place in such and such a place you will see a huge number of people going. It is just unfortunate that those prophets who claim to cure people do not even have the powers to do that. But because of our faith, we are driven by what we believe in, we are driven away from the drugs into those prophetic things. [Professional Healthcare Worker, Urban Facility, Southern Province].

R: Prayers, yes, and I tried taking holy water, anointing water. I used to take that knowing I am sick. I felt like, I felt it was keeping me [well] when I stopped treatment. I would use anointing water for cooking food.

I: Did you feel like anointing water was helping you?

R: It wasn’t helping as such, but putting everything in prayer in God’s hands knowing that God is putting his hands, there yes, I am his child he cannot leave me be. [Urban Female, Disengaged, Lusaka Province]

Such accounts suggest that ‘faith’–often articulated as ‘praying’–was one of several factors linked to self-efficacy, or lack thereof.

I have just put myself on prayers and encourage myself to pray to my father in heaven so that I can be taking my drugs. [Urban Female, In-Care, Eastern Province]

#### Social interaction

A strong and persistent theme that emerged in the accounts of PLHIV, HCWs and family members was the important role that family and close social relationships played in the problem solving processes relating to HIV care. These relationships cut both ways. For some, social relationships were a critical source of emotional and physical support, particularly when it came to helping with medication pick-ups:

I told my family. My husband was the number one, and then my family. I discussed it with my family […] I told them: “You know it’s best, because at times I may find I am not here. I may be somewhere but I will be able to call someone [in the family] and say that today is my date [for the clinic] you can go and get my medicine so that I don’t forget.

[Urban Female, Transfer, Lusaka].

I disclosed so that when I get sick and fail to wake up then I am able to send her to get me my medicine.

[Rural Female, In-Care, Southern Province].

However close relationships were not always a positive influence on treatment decisions. Previous literature has documented an association between non-disclosure and poor retention outcomes [23, 24]. In this study, many respondents described their fears that starting or staying in treatment would result in unintentional disclosure of their status, affect their valued social identity as a productive family or community member.

I haven’t told anyone [my status]. If, I was to tell even my sisters and my mother, they might start thinking that our brother is already dead, or maybe my child, it’s the end of him. That is the reason I haven’t told anyone.

[Rural Male, Disengaged, Eastern]

*I* thought my family members will start laughing at me or think I was just done for ….

[Rural Female, Disengaged, Western Province]

Others feared more material consequences, such as being ostracized or made destitute by their families.

R: The brother [-in-law] didn’t tell me. He was hiding it.

I: What do you think he was hiding it?

R: I don’t know the reason. I don’t know. Maybe he was scared […] that if he revealed it, that we might just [take away] our sister or something will happen at the house, something which is not supposed to happen.

[Urban Male, Next of Kin, Eastern Province].

For female respondents in particular, fear of disclosure arising from long-term treatment (including beyond the term of their pregnancy) was more often associated with materially damaging outcomes:

There is no one in the family who knows. I’m scared maybe they can be upset and leave me.

[Rural Female, Pregnant In-Care, Eastern]

Lay and professional healthcare providers also frequently described the impact that non-supportive spouses or family members had had on female patients’ decisions to start or stay in care.

These people, especially women who test in PMTCT, they can be tested without the knowledge of their spouses. Now for them to start treatment, and then to tell the spouse at home, it’s very difficult. So they can start that process, but on the way if they hear that their spouse can know, that person will stop coming for treatment.

[Rural Professional HCW, Eastern Province].

Such negative consequences were real for a number of respondents, as exemplified in the two quotes below:

I: Did you receive any support from your husband when you started treatment?

P: We are no longer together. We divorced when I got sick.

[Rural Female, In Care, Southern Province].

I: You mentioned that your family was not happy?

P: Yeah. I called my cousins and said: “Listen things are like this, and this this is my status so I am asking for help […].” So they said: “No you should stop treatment. Why are you taking medicine?” You know how families are. They think very soon we will find ourselves in problems. So that is what made them angry.

[Urban Female, Pregnant In-Care, Lusaka].

#### Motivational mechanisms

Models of health behaviour such as Bandura’s [25] often attribute behaviour change to motivational mechanisms such as perceived self-efficacy, outcome expectations and individual life goals (work, health and family). Analysis of data in this study suggests three key motivational mechanisms: self-efficacy relating to individual’s confidence to engage in ART; outcome expectations relating to individual’s desire to remain or become healthy, and key life goals focusing on normative family obligations.

#### Self-efficacy

Fear of HIV diagnosis and lack of confidence in relation to their ability to commit to or maintain life-long ART was a recurrent theme in the accounts of many PLHIV.

[My sister] was telling me to test […] But when I tested, I never told her that the results are [positive]. It was difficult for me to tell her. My heart is still not at peace. I am still upset about it. These were not my expectations.

[Rural Female, Eastern Province, Pregnant In-Care].

I: What is the reason why you were not drinking medication?

R: I was just scared and I had thoughts that said I can’t drink these medicines (ART).

[Rural Male, Transfer Eastern Province].

Poor understanding of HIV, and received wisdom in the form of myths and misconceptions about treatment were often mentioned in relation to this lack of confidence and an important barrier to seeking care.

I was scared. Like [if] I start medicine, what if one day I forget? Because I hear that when you start at 07: 00 hours, then it has to be 07: 00 [always]. If it is 12: 00, then 12: 00 always. So now I was asking, if let’s say it goes past ten minutes late? Maybe I am not around that time. So, if it is 12:00? If it goes ten minutes past 12:00, can that [taking ART] be done?

[Rural Male, Southern Province, Disengaged]

The main reason I didn’t start is […] The idea that when you start the drugs, you can’t stop until when you die. That is the very thing which was worrying. That when I started, I will be taking it every day and every day until such a time when my blood will be full of drugs.

[Rural Female, Pregnant In-Care, Eastern Province].

#### Outcome expectations

Nearly two-thirds (n = 55) of all patients interviewed stated that their engagement in HIV care and treatment was influenced by a desire to be healthy.

[I want to stay on ART] because I am HIV positive and I want to be healthy.

[Rural Female, Lusaka, Pregnant In-Care]

However, the desire to be healthy was almost always linked to respondents’ life goals. Responsibilities as a breadwinner or as a parent or care giver were particularly powerful motivators. Over a third of PLHIV interviewed mentioned that being healthy enough to look after their children was an important factor in decisions to seek treatment; this included some individuals who were not yet symptomatic and several in-care but ‘pre-ART’ respondents who expressed a desire to start treatment earlier in order to avoid getting too sick for the same reason.

I realized my children are still very young. So maybe I can be able to bring them up in the right way during the time [I] am still alive.

[Rural Male, In-Care, Southern Province]

It was my decision to start, because I knew that I’ll find myself in a [bad] situation. But now there is help [at the clinic], I can go for it. I see how I can take care of my children and my family.

[Urban Female, Transfer, Lusaka].

For pregnant women, protecting their child from HIV infection was similarly a powerful motivator for engaging in prevention of mother to child transmission (PMTCT) programs.

After they found me [HIV positive], they asked me whether I was free to start taking ARVs. Then I answered yes, because we need to protect the child. I decided it was better to save the child.

[Rural Female, Pregnant In-Care, Southern Province]

For post-natal mothers, however, a more mixed picture emerged regarding motivations to remain in care. As noted above, some women were fearful of disclosing their status to husbands or family, and absent the motivation to protect their baby from HIV infection, social interactions and interdependencies were described by respondents and HCWs as a barrier to ongoing engagement in care.

The mothers when they test they don’t disclose, most of them. Some … some will disclose to their partners. Others will not disclose. So you find that if a woman gets started on treatment they will not adhere, because the other partner doesn’t know what is going on.

[Professional Health Worker, Urban Site, Lusaka]

A further outcome expectation was the need to be physically capable of carrying out work and ‘not create troubles’ for the family were reported by many respondents as a key motivator for engagement in care:

[I was] getting more and more slim […] I had lost a lot of weight. So I told my sister-in-law and she told me to go to the clinic and get tested. She said: “Don’t be afraid because if you get afraid, you will put people in problems.” And it was true, because at that time I was really sick and I knew if I was scared of been tested and getting drug, I was going to put people in trouble.

[Rural Female, Transfer, Western Province].

In a number of cases, where ill-health was a real or potential barrier to being able to work, PLHIV were motivated to seek and remain in treatment to enable them to be sufficiently healthy to seek employment and lead productive lives.

Well, I enrolled because I thought that the day that I will get sick, I will not be able to work. I will just be sick like that. So I thought that in this village set-up where I live, if you just stay sick, there’s nothing that you can do. It’s better just to go to the clinic and get your medication. You can be fine and know how to take care of yourself.

[Urban Male, Pre-ART, Western].

For others, however, (particularly those who were asymptomatic) clinic visits that clashed with work commitments resulting in a trade-off between remaining in care and maintaining a livelihood. This was especially the case for several respondents whose jobs required mobility and clients in the early, intensive visit schedule mandated during the initial enrolment in ART.

When I just started [on treatment], they used to make me come every two weeks for review. Then at work, they started swapping me because I was on contract, and if a client is not happy with your services they would ask for another person. So the boss would swap me with someone else. And in the end, the boss decided that despite wanting to keep me on, the clients were complaining, so that is how they gave me a month’s notice to leave the job.

[Urban Male, Transfer, Southern]

#### Problem solving

Comparatively few problem solving strategies were highlighted by respondents. Those described tended to further highlight the importance of individual’s social networks in influencing choices about engagement in care. A key example related to decisions to transfer clinic, an action described by 11 ‘transfer’ respondents as a way to ameliorate various structural (e.g. distance related), social (concerns about stigma and identification) and motivational (degree of psycho-social support from individual HCW) barriers experienced.

Going for a transfer elsewhere [can happen due to] a number of problems which we encounter. Maybe the people who are looking after you [at home] say they are moving and going to stay elsewhere. Or, us people who are sick, there are times when we still have work in far places

[Rural Male, Transfer, Eastern Province]

I started treatment but transport was difficult for me to find so I left and went to another clinic that is where I started getting treatment from. But again life became hard so I came back to my original place, where transport was less difficult.

[Rural Female, Transfer, Lusaka Province]

As noted in previous sections, disclosure also constituted a problem solving strategy for some patients, since it enabled them to access emotional or material support to continue accessing care.

In relation to the structure of HIV services, some participants noted the importance of ‘knowing your place’ and ‘just sticking to the rules’ when it came to receiving good care.

We come here [to the clinic] to get medication. We follow the laid down rules at the clinic so that we can be helped […] Even if you are feeling better, you will have to continue because those are the rules of the clinic. And when they tell you that you are supposed to come on the seventeenth (of the month) you have to go there […] and they will tell you: “Here are the drugs”. And you have to thank them and ask when should I come here some other time, and they give you [the date] just like that. So I am just following what the clinical rules are, am in the hands of the clinic

[Rural Male, In Care, Eastern Province]

Finally, we note that some problem-solving strategies may be hidden at first sight. For example, managing disclosure is a problem-solving strategy to secure social support and care in the local context.

### Action state

#### Social interdependence

Close social relationships are those in which the ‘action scripts’ of people involved are intertwined. A common example of a close social relationship would be a married or cohabiting couple; each individual in the relationship has the ability to help, or hinder the other’s choices and actions and thus affect their ability to achieve goals related to love, work or self-care [21]. The vast majority of persons living with HIV interviewed during this study were married (71%) and analysis confirmed a high degree of social interdependence among married and cohabiting couples guiding decisions about seeking, or remaining in care. For example, many participants described their partners’ orientation towards treatment or capability to provide support as an essential influence (positive or negative) on decisions to test, enroll and remain in care.

We are just encouraging one another [in our treatment] and I really thank God for my husband because he really encouraged me not to lose hope.

[Rural Female, Pregnant In-Care, Lusaka Province]

It’s because I have started the treatment but my husband has not started and that is what is paining me, I ask myself, “Why?” I would get upset but on the other hand, I would cool down, Maybe it’s because of the pregnancy. So, I tell myself that. “It’s okay. Let me protect my child.” But him! He says, “I can’t start now!”

[Rural Female, Pregnant in Care, Eastern Province]

#### Health outcomes

Many patients and most notably those still in-care, described physical recovery following treatment as an important driver of decisions to remain engaged in care. Experiences ranged from generalised statements regarding ‘having more energy’ to more detailed accounts outlining ‘Lazarus-like’ recoveries.

[I’m glad I am on treatment] because taking the ARVs it’s different compared with a long time ago. When I wasn’t taking any ARVs, during that time I use to have coldness, right now there is nothing like that.

[Rural Female, Lusaka Province, In-Care]

When I was sick I was not well because I had no strength, I wasn’t even able to walk. But from the time I started medication, my strength came. I wasn’t even eating [before] but I started eating, that is how I felt, I changed and my strength was restored

[Rural Female, Southern Province, Transfer].

Conversely, long term engagement was negatively impacted among (a very few) respondents who experienced intense side effects, or, physical symptoms that participants associated with HIV treatment. Many patients reported initial treatment side effects that were attenuated after a short period. But for a few, side effects were more intense or of longer duration and played a role in decisions to disengage.

I started taking ART which didn’t work for me. The moment I [took it] I would experience heart palpations, headaches and painful legs. I took them until I delivered then when the baby was one year or one year and something, then I stopped. The moment they finished I stopped feeling [those symptoms].

[Urban Female, Southern Province, Disengaged].

At first it used to make me feel dizzy. Like on my part it used to make me dizzy then it would take away my appetite. I used to fail to eat but I was on medication, I used to fail to eat. I was feeling weak.

[Urban Female, Lusaka Province, Disengaged]

Notwithstanding these accounts, most respondents in this study suggested that the influence of side effects on their decisions to remain engaged once in care was limited, due to a combination of counselling preparation and the relatively short duration of symptoms.

## Discussion

This study represents a rigorous examination of the complex interplay between contextual, social and motivational forces driving the decisions of Zambians living with HIV regarding engagement in HIV care and treatment. Grounded in mixed qualitative methods that included interviews with patients and family members, focus groups with health workers and direct observations in the clinic setting, we were able to triangulate data and examine how different factors contributed to engagement outcomes across a geographically representative sample of Zambians living with HIV.

Overall, the data fit SAT theory well, with particular synchronicities between the theory’s propositions around the Action Contexts—physiological state, social setting and service settings and their links to social interactions and motivations. Table 1 summarises a series of findings about the impact of contextual factors on self-change processes and individual actions in the Zambian setting. This table provides a starting point, helping to identify, for example, a strong feedback loop between people’s experience of ill-health as a key action context, their desire and expectation of improved health as a motivation for self-change, and the decision to engage in care and subsequently treatment effects. The findings demonstrate, for example, how the patients were motivated to seek care as a means to an end—such as earning livelihoods or leading productive lives but mediated by perceived and realized material and emotional support from their social networks. The desire to resume a productive life was often not supported in the context of both inflexible workplaces and often inflexible clinic hours. In such contexts, people well enough to lead productive lives were less likely to engage in care; those too ill, were more likely to initially engage in care but then discontinue if the care did not allow them to return to work—unless they had emotional and material support from their social networks. Thus, systematic analysis using SAT helped move beyond a ‘compartmentalised’ interpretation of data to more holistic view by providing the lenses needed to integrate and understand the social/environmental and individual/behavioural factors at play when patients changed from one state to another in the HIV continuum.

**Table 1:** Key factors influencing engagement in care and treatment among Zambian persons living with HIV.

Theme	Sub-Theme	Key Finding	Influence on Engagement in Care
**CONTEXTUAL**	Physiological State	Experience of illness	**Facilitator–**catalyst to seek help; influences motivation by placing life goals or other projects in danger
	Broader Social Setting	Community attitudes and perceived stigma around HIV	**Barrier**–persistent perceptions of HIV-related stigma and gossip outside the family unit; embodied in fears of being written off as ‘promiscuous’ and or ‘useless’
	Health System	Infrastructure / Location / Opening Hours	**Barrier**–chronic congestion undermines confidentiality undermining self-efficacy & motivation; long distances to travel and waiting times create opportunity costs for accessing care that impact on livelihoods
		HCWs as trusted sources of information / mentors HCW with weak service values and respect for clients	**Facilitator–**empathetic treatment and counselling provided by some HCW improves generative capacity (knowledge & understanding) and problem solving capacity of some HIV-positive individuals **Barrier–**disrespect and abuse by some HCW undermines self-efficacy and reinforces concerns relating to social stigma;
		Standardised diagnostic processes / tests	**Facilitator–**strengthens expectation of achieving improved health via perceptions of receiving rigorous and appropriate treatment
	HIV Service Delivery	Free services	**Facilitator**–reduces out of pocket expenses associated with HIV care; eases reliance on extended social network and can contributes to perception of self-efficacy.
		Targeted HIV services /(couple counselling; etc)	**Facilitator–**perceived as service sensitive to the socially-complex needs of PLHIV access, help to motivate engagement via improved understanding, improved self-efficacy and problem solving strategies.
		Inflexible HIV visit and medication guidelines	**Barrier**–perceived as insensitive to the socially-complex lives of persons with HIV; undermines sense of self-efficacy and patient-provider trust;
**SELF-CHANGE PROCESSES**	Generative Capabilities	Knowledge / understanding of HIV/AIDS and ART	**Facilitator–**improved understanding of what HIV is, and how ART works improves self-efficacy and problem solving capacity
	Social Interactions	Disclosure / non-disclosure to family and friends	**Facilitator (Disclosure)–**Enables PLHIV to draw on emotional and material support from social network **(Non-disclosure) Barrier–**Indicative of lack of confidence in close social networks to provide support; non-disclosure makes accessing support to remain engaged difficult **Gender roles–**economic and financial vulnerability of some women makes disclosure less likely
	Motivations	Self-efficacy—Personal acceptance / denial of status	**(Acceptance) Facilitator–**promotes confidence to take direct action vis. seeking/remaining in HIV care **(Denial) Barrier–**contributes to reluctance / fear of addressing the problem directly
		Expectations & Goals: staying healthy; looking after children; remaining employed	**Facilitator–**PLHIV want to protect health in order to fulfil socially valuable goals; key motivation in attempts to get and stay healthy **Barrier**–Engaging in care sometimes endangers fulfilment of goals (e.g. employment) due to time/cost/structure of clinic visits; or due to risk of social consequences.
**ACTION STATE**	Social Inter-dependency	Spousal influence/orientation to HIV care	**Facilitator**–direct encouragement/permission from life partner improves material & psychological capacity to seek care **Barrier**–experience of, or anticipated resistance from life partner inhibits willingness / capacity to seek care
	Outcomes	Impact of side effects / physical symptoms	**Barrier–**associated with negative treatment experience undermining commitment to stay in care
		Experience of recovery	**Facilitator**–strengthens trust in care, clinic services improves physical capacity to attend clinic **Barrier**–contributes to apathy/resentment relating to ongoing clinic visits including due to competing life events/activities.
		Experience of illness (again)	**Facilitator–**catalyst to seek help, (re)activates desire to be healthy

We also found a good fit between our data and the first three self-change processes (generative capabilities, social interactions, motivations) but comparatively less robust data relating to problem solving processes, alone or in conjunction with the other three self-change processes. The one obvious exception related to (in care) patients’ decisions to adapt their own schedules and needs to clinic schedules in order to ensure continued access to ART. Further work is needed to better theorise action states along the HIV care continuum and the cyclical nature of health seeking behaviour within an evolving context and self-changing processes. For instance, how do self-changing processes and action states shift as HIV care moves towards differentiated service delivery disaggregated by patient characteristics such as age, marital status, risk of transmission and stability on ART? Thus, our findings represent tentative ‘context-mechanism-outcome’ configurations [26–28] that could and should be validated through further research to inform our understanding of which contexts influence which mechanisms of change.

In the following discussion, however, rather than focusing on series of context-mechanism-outcome configurations, each with multiple contingent factors, we draw on previous conceptual work in this area [29] and our own analysis and experience to posit a set of higher-order propositions about the nature of engagement in HIV care, in Zambia and beyond. We do this recognizing that complexity typically emerges from what appear to be ‘simple’ interactions and rules [29–31] and that the most robust and efficient conceptual models are often those that focus on a small number of strong statements about behavioural and systems dynamics [29].

### Illness and healthcare utilization are social experiences

Findings from this study strongly suggest that the experience of living with HIV and engaging in HIV care (or indeed any health care) are social—not individualistic—experiences. Data presented in this paper demonstrates, for example, how varying levels of social interaction and interdependence mediated individual’s willingness (motivation) and capacity (knowledge and problem solving skills) to engage in care. Broad social norms relating to family life were frequently expressed as individual motivations such as the goal of looking after or protecting children. These were, in turn, reinforced by respondents’ perceptions about families’ and friends’ expectations of them. Mirroring findings from multiple other settings [32–34] data from this study consistently pointed to people’s fear of being ‘laughed at’ if they were widely known to be living with HIV. Indeed, fear for their identity as a productive member of society led a number of respondents to avoid disclosing to family members as they did not want to ‘worry’ or ‘upset’ them, or be ‘thought of as being dead’.

The highly gendered nature of engaging in HIV care was also clear from women’s and health workers accounts, which consistently flagged social (stigma) and economic consequences (abandonment; destitution) for women accessing care with similar experiences documented elsewhere in the region [35–37]. Considering these multiple and often-overlapping consequences was part of a wider assessment of the relative costs and benefits of being engaged in care. It demonstrates how a seemingly simple decision is governed, to a large extent, by a broader and socially sanctioned set of expectations and norms that guide social interaction. To the extent that engagement in care helped individuals achieve or recapture socially sanctioned life goals or meet these expectations, such norms were a facilitator for engagement in care. But where engagement in care threatened these expectations and normative goals, it was de-prioritised.

### Patients and their networks tend to act collectively

Related to the first, a second overarching finding from this study is that patients and their networks tended to act collectively to negotiate and navigate HIV care. Reflecting concerns documented in many other settings [20, 24, 38–40], Zambians living with HIV in this study stressed both the physical and psychological burdens of living with HIV and (independently of that) accessing care. Our respondents described the struggle to manage the array tasks expected of them (e.g. making and keeping appointments; eating well; adhering to medication schedules), which occurred alongside the demands of their everyday life. Many described how, as those burdens accumulated, their ability to remain in care was dependent on the support they received from friends and family. Indeed, capacity to engage in care was frequently contingent on the strength and reach of social networks. Although most respondents in this study described having some kind of mutually supportive social relationships, the intensity, size and complexity of those connections differed. In keeping with findings from elsewhere [41–44], participants in this study (pre-dominantly those still in care) described a strong supportive social network to whom they had disclosed their HIV status. For these individuals, the aim of disclosing was to access material and emotional support that help them remain in care. Conversely, more of those who had disengaged from care described seeking care as difficult due to lack of support—financial, physical or psychosocial—or in the case of women, due to the perception that being in HIV care would actually threaten that support. Intuitively, moreover, the less support available from a participants’ immediate social network the more important the prevailing conditions at the health centre and emotional support available from health care workers at that clinic became. Lay and professional health workers thus form an intrinsic part of individuals’ support network and their responsiveness to patients’ needs influence patients willingness to engage and capacity to remain in care.

These findings challenge an implicit assumption about the central role of individual agency that underpins many studies seeking to explain engagement, adherence and retention in HIV care [45–54]. Rather than the individual being a key ‘unit of analysis’, our findings suggest that in settings such as Zambia, it is in fact groups of people, whose beliefs, actions, resources and networks help define and compensate for variations in individual situation and capacity [29] that represent a more meaningful starting point for enquiry. Such findings suggest moreover that much more focus must be placed on social programs and policies targeting local communities’ understandings of HIV and HIV care, in order to counter negative perceptions and the associated social pressures to disengage from treatment.

### The capacity of patients and their networks to negotiate care is influenced by the rules and structure of health and HIV services

A third overarching finding from the current research relates to the way the rules and structures of HIV services specifically, and the health system more generally, impact on access and engagement in HIV care. In Zambia, we found HIV care and treatment services to be underpinned by a range of managerial and behaviourial expectations that emphasized self-care, self-efficacy, and engagement. These expectations were often difficult for individual patients to meet, combined as they were with frequently geographically distant or overcrowded health centres. *Prima facie*, these findings are not new, since many studies have previously explored the role of health system and service-delivery factors in retention in care [15, 55–57]. In combination with the two findings outlined above, though, this study extends our understanding by considering them explicitly through the lens of engagement in care as a collective action and a social experience.

Our findings show that where the Zambian health system was more closely aligned with the social nature of health service use—including the provision of psychosocial counselling, opportunities for couples testing, and investment in community outreach and follow-up services—patients reported facilitation of both their initial and continued engagement in care. Conversely, respondents described how certain features of location, environment or service-delivery model that worked against long-term engagement. For example, many expressed concerns about lack of physical privacy in the clinic, which combined with fears about unintentional disclosure and stigma in the community impacted on their confidence to remain in care. Many others reflected on the rigid treatment guidelines and rules about medication pick-up and missed visits, declaring these rules to be highly insensitive to the social and economic obligations of day-to-day life. The need for revision to both the physical resourcing and service delivery models being used is thus evident.

May et al [29] among others [58–61] suggests that experiences of health system or service-related constraints can reinforce or change patient behaviours. Our data supports this intuitive proposition and point to several areas in which reform of HIV service arrangements could improve overall responsiveness to patients’ socialized needs for long term engagement in care. Data suggest, for example, that the current practice of ‘intensive adherence’ following a missed visit, which effectively conceives of patients as individualistic and health-maximizing beings, may ultimately be counter-intuitive to the aim of improving long term adherence and retention in care. Rather, clinical guidelines and health worker practice must be flexible to account for the social lives of those seeking care, and the multiple but often inconsistent factors that will affect their ability to access care over a lifetime. These factors require the health system to be able to better tailor service intensity along different lines, including type, location, provider and frequency [62].

Given the substantive role of health workers as part of patients’ social support network and their influential role in decisions about staying in care, data also point to the need for revisions to upstream leadership policy, work incentives and health worker socialization, if sustained improvements in health worker attitude, orientation and responsiveness are to be achieved [61, 63]. Although not the explicit focus of this work, it also follows that interventions that build and strengthen stronger social networks for PLHIV who are struggling to engage, and that equip them to more effectively navigate inevitable system constraints (as demonstrated by positive accounts of the impact of community-based service models and demands for more community-based education) will likely improve long-term engagement in care. Our interpretation aligns with the conclusions of several recent reviews examining what sort of interventions have consistently improved retention in HIV care across settings [14, 64].

## Conclusion

Leichow and Milstein [65] write: “We must guard against the tendency to acknowledge the presence of complex relationships […] while employing analytic methods or program practices that exclude or assume independence among those that are included.” Yet in the literature focusing on HIV adherence and retention in LMIC, there has been a tendency to do just that. Building on a number of key examples of research that has sought to engage with, rather than reduce such complexity, the current study moves beyond a listing of barriers and facilitators and clustering factors in traditional socio-ecological groupings to provide a more robust explanatory framework for the way different environmental, social, biophysical and motivational factors interact and influence each other to produce HIV care engagement outcomes. Our findings highlight the social nature of illness and healthcare utilization for HIV and potentially other chronic diseases in Zambia, the importance of collective action in accessing and remaining in HIV care that should underpin future efforts to revise and reform the systems and structures of the health system.
